# Clinical heterogeneity and diagnostic challenges in *CASK*-related neurodevelopmental disorders: a longitudinal observational study

**DOI:** 10.3389/fpsyt.2026.1804756

**Published:** 2026-07-13

**Authors:** Iliyana Hristova Pacheva, Elena Timova, Tihomir Todorov, Iglika Sotkova, Katerina Gaberova, Ralitza Yordanova, Tzvetelina Tzvetanova, Fani Galabova, Nevyana Ivanova, Albena Todorova

**Affiliations:** 1 Department of Pediatrics, Medical University of Plovdiv, Plovdiv, Bulgaria; 2 University Hospital “St George”, Plovdiv, Bulgaria; 3 Genetic Laboratory Genika, Sofia, Bulgaria; 4 Molecular Medicine Center, Department of Chemistry and Biochemistry, Medical University of Sofia, Sofia, Bulgaria

**Keywords:** autism spectrum disorder, *CASK* gene, cerebral palsy-like phenotype, epilepsy, growth retardation, intellectual disability, microcephaly, neurodevelopmental disorder

## Abstract

**Background:**

Pathogenic variants in the *CASK* gene cause a broad spectrum of X-linked phenotypes ranging from microcephaly with pontine and cerebellar hypoplasia (MICPCH) to only mild intellectual disability (ID). Variable clinical pictures pose significant diagnostic challenges.

**Methods:**

We conducted a retrospective observational study with longitudinal follow-up at a tertiary pediatric neurology center over a 10-year period (6,179 patients were hospitalized and evaluated). Since 2015, genetic testing using next-generation sequencing gene (NGS) panels, including *CASK* gene, was performed in patients with MICPCH and the first patient was confirmed through hereditary ataxia NGS panel. Two additional patients were identified among 105 children with suspected genetic neurodevelopmental disorders undergoing CentoNeuro panel (including 1,902 genes), which was available during a period (2023–2024). Clinical, neuroimaging, and genetic data were analyzed during follow-up.

**Results:**

Two female patients carried *de novo* loss-of-function *CASK* variants and presented with MICPCH, progressive developmental impairment, abnormal muscle tone, postnatal growth retardation, and epilepsy in one case. The male patient carried an inherited likely pathogenic missense variant and exhibited severe ID, drug-resistant epilepsy, autistic features, and a cerebral palsy–like phenotype without microcephaly or pontocerebellar malformations. All patients demonstrated periods of developmental arrest or regression, suggesting non-linear developmental trajectories. Marked intrafamilial phenotypic variability was observed.

**Conclusions:**

*CASK*-related disorders may present with severe neurodevelopmental impairment and cerebral palsy–like phenotypes, even in the absence of characteristic neuroimaging findings. A cerebral palsy-like phenotype, postnatal growth retardation, and variable ID should raise suspicion for *CASK*-related disorders. Comprehensive genetic testing, including next-generation sequencing, is essential for accurate diagnosis.

## Introduction

Pathogenic variants in the *CASK* gene are a recognized cause of a broad spectrum of X-linked and X-dominant neurodevelopmental disorders (NDDs), characterized by marked clinical and genetic heterogeneity (1–3). The phenotypic spectrum ranges from severe neurodevelopmental impairment with microcephaly and pontine and cerebellar hypoplasia (MICPCH) to milder forms of intellectual disability without structural brain abnormalities (3–5). Variable expressivity, age-dependent progression, and sex-specific differences substantially contribute to diagnostic complexity (6).

The *CASK* gene encodes a calcium/calmodulin-dependent serine protein kinase that plays a critical role in synaptic development, neuronal migration, and brain maturation (4, 7). Loss-of-function variants, particularly in females, are most commonly associated with MICPCH, whereas missense variants, especially in males, may result in a wide range of phenotypes, including intellectual disability, epilepsy, motor impairment, and ocular abnormalities such as nystagmus or optic atrophy (1, 6). Notably, affected males may present without microcephaly or cerebellar hypoplasia, further complicating clinical recognition (8).

Epilepsy is a frequent but variably expressed feature of *CASK*-related disorders, with reported onset ranging from infancy to adolescence and seizure types including epileptic spasms, focal seizures, and generalized seizures (3, 5, 8, 9).

Postnatal growth retardation, autistic features, and cerebral palsy–like motor phenotypes have also been described (2, 3). The absence of a pathognomonic clinical profile often leads to delayed diagnosis, particularly in patients lacking characteristic neuroimaging findings (1).

Since the initial description of *CASK*-related disorders, approximately 180 affected individuals have been reported worldwide, as summarized in cohort studies and comprehensive reviews (1–3, 5, 6). However, the true prevalence is likely underestimated, particularly in individuals with milder or atypical presentations. The increasing availability of next-generation sequencing (NGS)-based diagnostic approaches has substantially improved detection of pathogenic *CASK* variants beyond classical phenotypes (10, 11).

In this study, we report the first cases of *CASK*-related neurodevelopmental disorders from Bulgaria. By describing three pediatric patients diagnosed through different genetic testing pathways and exhibiting intra- and interfamilial phenotypic variability, we aim to expand the clinical spectrum associated with *CASK* variants and highlight the diagnostic challenges encountered in routine clinical practice.

## Methods

### Study design

This study was designed as a retrospective observational study with targeted case identification of *CASK*-related neurodevelopmental disorders and longitudinal clinical follow-up. Patients were evaluated at the Pediatric Neurology Ward, Department of Pediatrics, University Hospital–Plovdiv, a tertiary referral center, over a 10-year period (2016–2025).

### Patient ascertainment

During the study period, 6,179 pediatric patients with neurological disorders were hospitalized and evaluated at the department. Since 2015, patients presenting with clinical features suggestive of MICPCH, possible *CASK*-related disorders were systematically evaluated, and genetic testing using next-generation sequencing (NGS) gene panels, including the *CASK* gene, was performed when clinically indicated.

The CentoNeuro panel (Centogene GmbH; 1,902 genes, including CNV analysis; see **Supplementary Table 1**) became available during a later 2-year period (2023–2024) and was applied to a selected subgroup of patients with suspected genetic neurodevelopmental disorders, including developmental delay/intellectual disability, autism spectrum disorder, epilepsy, and cerebral palsy–like motor phenotypes of unclear etiology.

### Clinical evaluation

Clinical evaluation included detailed physical and neurological examinations, as well as assessment of developmental status. Neurodevelopmental status was assessed using age-appropriate clinical and neuropsychological instruments, including the Developmental Profile II and the Stanford–Binet test. Autism spectrum disorder was diagnosed according to DSM-5 criteria (12).

Electroencephalography (EEG) and brain magnetic resonance imaging (MRI) were performed when clinically indicated. Additional investigations, including metabolic, endocrine, and ophthalmological assessments, were carried out depending on the clinical presentation.

### Genetic analysis

Genetic testing was performed using next-generation sequencing (NGS) –based approaches, including targeted gene panels and, where applicable, whole-exome sequencing with integrated NGS-based copy number variation (CNV) analysis. All pathogenic and likely pathogenic *CASK* variants identified by next-generation sequencing were confirmed by Sanger sequencing. Parental testing was performed in all cases.

Sequence data were analyzed using standard bioinformatic pipelines. Variant interpretation was conducted according to standard criteria, including ACMG guidelines, and supported by available databases such as ClinVar. Variants were classified as pathogenic or likely pathogenic based on established criteria.

### Ethical considerations

The study was conducted in accordance with the principles of the Declaration of Helsinki and was approved by the Scientific Ethics Committee of the Medical University of Plovdiv. Written informed consent for genetic testing and data use was obtained from the parents or legal guardians of all patients. All data were analyzed anonymously.

## Results

Three patients with *CASK*-related disorders were identified during the study period. Patient 1 was genetically diagnosed in 2020 following strong clinical suspicion of a cerebellar disorder and underwent targeted next-generation sequencing using a hereditary ataxia gene panel. During a subsequent 2-year period (2023–2024), when the CentoNeuro NGS panel became available in our pediatric neurology practice, two of 105 patients undergoing testing were found to carry pathogenic or likely pathogenic *CASK* variants (Patients 2 and 3). Parental testing confirmed *de novo* variants in Patients 1 and 2, while extended family testing in Patient 3 revealed X-linked inheritance with marked intrafamilial variability.

### Patient 1

Patient 1 is a 9-year-old girl with a *de novo CASK* frameshift variant, NM_001367721.1:c.1279dupG (p.Glu427Glyfs*24). She was born at term after an uneventful pregnancy (birth weight 2,980 g; length 48 cm; head circumference 33 cm). Newborn hearing screening was normal.

From early infancy, progressive postnatal microcephaly and failure to thrive became evident. At 6 months of age, she was referred due to insufficient head growth and poor feeding. At this age, she reached for toys, rolled from supine to prone, did not sit independently, cooed but did not babble, and laughed aloud (her neurodevelopment was considered borderline, but later followed a non-linear course). At 1 year of age, she could babble, responded inconsistently to her name, visually tracked objects, and showed interest in them. She received regular physiotherapy with improvement, achieving independent sitting and transition to sitting at 1 year and 4 months. At 1 year and 6 months, she could pull to stand, follow elementary commands, and babble; her developmental quotient indicated mild neurodevelopmental delay.

A transient regression occurred, including loss of babbling and reduced social attention, followed by clear developmental progress between 2 and 3 years of age. During this period, she demonstrated babbling, followed some commands, had an expressive vocabulary of 5–6 words, and showed improvement in motor skills, including assisted ambulation.

However, after 3 years of age, a second phase of developmental stagnation and regression occurred, predominantly affecting social and emotional domains. Autism spectrum–related features emerged, including reduced social engagement, absence of speech, inability to follow even simple commands, stereotyped behaviors, restricted interests, and impaired adaptability. After this period, neurodevelopmental arrest became evident.

Neuroimaging demonstrated pontocerebellar hypoplasia, initially identified on CT at 6 months of age and confirmed by MRI at 11 months, consistent with the MICPCH spectrum.

Epilepsy onset occurred at 4.5 years of age, presenting as clusters of epileptic spasms. Subsequent sleep EEG showed epileptiform activity consistent with modified hypsarrhythmia. Seizures remained pharmacoresistant despite multiple antiseizure medications, and treatment with ACTH or steroids was declined by the parents.

Muscle tone abnormalities were prominent and dynamic. During infancy, increased tone and spasticity of the limbs were observed, accompanied by intermittent athetoid movements, consistent with a mixed cerebral palsy–like phenotype. Spasticity was most pronounced early in life and partially improved over time, allowing acquisition of assisted gait.

At the latest follow-up, she exhibited severe to profound intellectual disability, persistent autistic features, progressive microcephaly, and marked postnatal growth retardation.

Alternating convergent strabismus was noted. No clinical suspicion of hearing loss was present; however, auditory evoked potentials could not be performed.

### Patient 2

Patient 2 is a 4.5-year-old girl with a *de novo* pathogenic *CASK* nonsense variant, NM_001367721.1:c.79C>T (p.Arg27*), identified using the CentoNeuro NGS panel and confirmed by Sanger sequencing (absent in both parents and a sibling). She was born at term after an uncomplicated pregnancy (birth weight 2,920 g; length 49 cm).

Global developmental delay and progressive postnatal microcephaly were noted at 1 year of age. She achieved sitting at 7 months and standing at 13 months. Independent walking was markedly delayed; at approximately 3.5 years of age, she was able to take only a few steps, and at 4.5 years her gait remained unstable.

A mild regression in expressive language was reported around 4 years of age, including loss of babbling and early vocalizations, followed by partial recovery with return of babbling and two expressive words. Motor stereotypies were observed; however, social interaction remained preserved, and formal diagnostic criteria for autism spectrum disorder according to DSM-5 were not met.

Muscle tone abnormalities showed a fluctuating course. Neurological examinations documented predominantly axial hypotonia, which improved over time, along with transient mild dystonic movements and brisk reflexes, resembling an ataxic cerebral palsy–like phenotype at 4 years of age.

Growth retardation had been documented since 1.5 years of age.

Brain MRI demonstrated cerebellar hypoplasia within the MICPCH spectrum. No seizures were observed, and EEG remained normal during follow-up. Auditory evoked potentials were normal.

### Patient 3

Patient 3 is a 16-year-old boy who presented with global developmental delay, first evaluated at 1 year and 6 months of age as moderate developmental delay, evolving into profound intellectual disability (IQ <20) by 5 years of age.

He rolled from supine to prone at 7 months of age, achieved independent sitting at 2 years and 5 months, and stood with support at 5 years of age. He had no speech, only non-articulated sounds, did not follow commands or visually track objects, and lacked social communication. From early childhood, he exhibited multiple stereotypies, including rhythmic rocking and eye squeezing, as well as other stereotyped behaviors, and was considered to have autism spectrum disorder.

Epilepsy developed at 7 years of age, initially presenting with generalized tonic–clonic seizures and later evolving into a mixed seizure phenotype with both focal and generalized events, which became drug-resistant over time. Serial EEGs demonstrated progressive abnormalities, evolving from focal epileptiform discharges to multifocal and generalized spike–slow wave activity.

Brain MRI revealed nonspecific white matter gliotic foci without pontine or cerebellar hypoplasia.

Postnatal growth retardation was evident, with short stature, delayed bone age, and underweight. Endocrine evaluation revealed low insulin-like growth factor 1 (IGF-1) levels with normal thyroid function.

Ophthalmological examination demonstrated mildly pale optic discs, consistent with optic disc subatrophy, and alternating convergent strabismus.

Motor findings were consistent with a mixed cerebral palsy–like phenotype. He exhibited axial hypotonia with variable limb tone, predominantly increased, hyperreflexia, contractures, and severe motor impairment, with inability to walk independently (GMFCS level IV). Intermittent dystonic–athetoid movements were observed, supporting a dynamic neurodevelopmental disorder rather than a static perinatal motor injury.

Genetic testing identified an inherited *CASK* missense variant, NM_001367721.1:c.1466G>A (p.Arg489Gln), currently classified as likely pathogenic following reclassification from a variant of uncertain significance. The variant was also identified in additional family members, including the patient’s mother, maternal grandmother, and maternal uncle, consistent with X-linked inheritance.

Family evaluation revealed marked intrafamilial phenotypic variability. The female relatives had no prior diagnoses; following neurological and psychological assessment (Wechsler Intelligence Scale), they were found to have mild or borderline intellectual disability with normal neurological examinations and no microcephaly. The patient’s mother had mild intellectual disability, and the maternal grandmother had borderline IQ to mild intellectual disability. The surviving 37-year-old uncle had previously been diagnosed with moderate intellectual disability; however, reassessment revealed mild axial hypotonia, preserved ambulation with a clumsy gait, no microcephaly, and no epilepsy up to that age. No medical records were available for the deceased uncle; according to maternal history, he did not have microcephaly, had a cerebral palsy–like phenotype, was able to sit but not stand, and had frequent daily generalized tonic–clonic seizures beginning in the first year of life. He died at 4 years of age during an episode of status epilepticus, without available genetic testing. None of the relatives had undergone brain CT or MRI.

A comparative summary of clinical features, neuroimaging findings, developmental course, and genetic results of three patients is provided in **Table 1**.

**Table 1:** Clinical, neuroimaging, and genetic characteristics of patients with *CASK*-related disorders.

Feature	Patient 1	Patient 2	Patient 3
Sex	Female	Female	Male
Age at first presentation	6 months	1 year 5 months	1 year 5 months
Age at last follow-up	9 years	4 years	16 years
*CASK* variant	c.1279dupG (p.Glu427Glyfs*24)	c.79C>T (p.Arg27*)	c.1466G>A (p.Arg489Gln)
Variant type	Frameshift (loss-of-function)	Nonsense (loss-of-function)	Missense (likely pathogenic)
Mode of inheritance	*De novo*	*De novo*	X-linked inherited Positive (5 affected relatives; multigenerational family)
Genetic testing method	Targeted genetic testing Hereditary ataxia NGS panel (clinical indication)	CentoNeuro NGS panel	CentoNeuro NGS panel
Sanger confirmation	Yes	Yes	Yes
Parental testing	Yes	Yes	Yes
Head circumference	Progressive postnatal microcephaly	Progressive postnatal microcephaly	Normal range
Growth retardation	Yes (severe)	Yes	Yes
Developmental course	Arrest → partial progress → regression → arrest	Mild regression → partial speech recovery → progress in motor skills and social communication	Progressive impairment at early childhood → static
Intellectual disability at 1 year of age	Mild	Mild	Moderate
Intellectual disability at last follow-up age	Profound	Moderate	Profound
Muscle tone abnormalities	Axial hypotonia, limb spasticity, dyskinesia (mixed CP mimic)	Mild hypotonia, (atactic CP mimic)	Axial hypotonia, variable limb tone, dystonia (mixed CP mimic)
Motor function	Assisted ambulation only	Unstable walking	Non-ambulatory (GMFCS IV)
Autistic features	Yes	No (Stereotypies only)	Yes
Epilepsy	Yes (epileptic spasms; drug-resistant)	No	Yes (focal and generalized tonic–clonic seizures; drug-resistant)
Neuroimaging	Pontocerebellar hypoplasia (MICPCH)	Cerebellar hypoplasia (MICPCH)	No PCH; nonspecific white matter lesions
EEG findings	Modified hypsarrhythmia	Normal	Multifocal and generalized epileptiform discharges
Ophthalmological findings	Alternating strabismus	None significant	Strabismus, optic disc pallor

CP, cerebral palsy; EEG, electroencephalography; GMFCS, Gross Motor Function Classification System; MICPCH, microcephaly with pontine and cerebellar hypoplasia; NGS, next-generation sequencing; MRI, magnetic resonance imaging.

**Figure 1:**
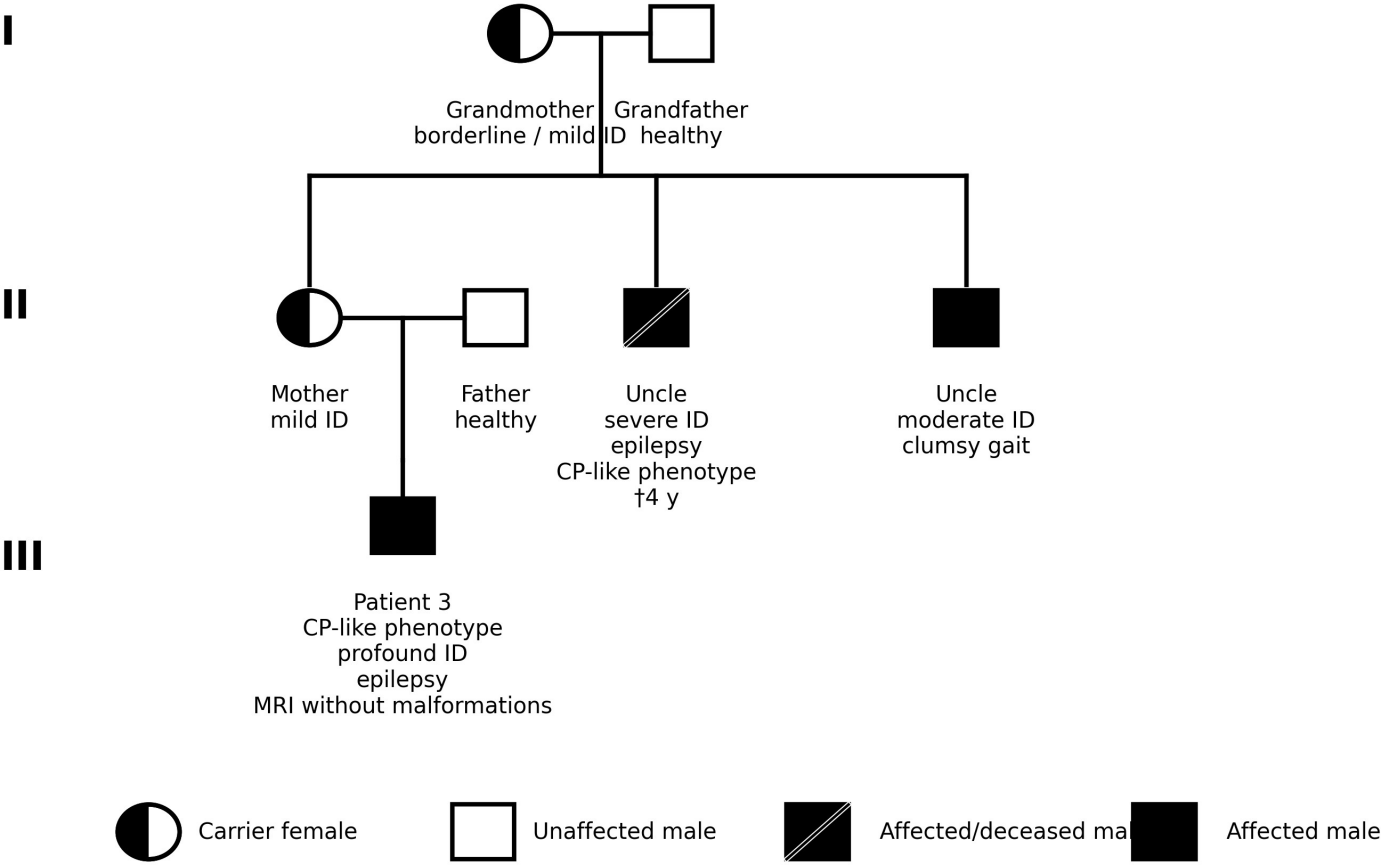
Pedigree of patient 3’s family demonstrating X-linked inheritance of the CASK variant c.1466G>A (p.Arg489Gln). The proband (Patient 3) is indicated in generation III.

The pedigree is presented in **Figure 1**.

## Discussion

This case series, including three index patients and four additional affected family members from Patient 3’s family, illustrates the broad phenotypic spectrum of *CASK*-related neurodevelopmental disorders and highlights the substantial diagnostic challenges posed by their clinical heterogeneity. Our findings confirm that *CASK* variants may underlie a wide range of clinical presentations, from classical MICPCH to severe neurodevelopmental impairment without typical pontocerebellar hypoplasia, and even to mild intellectual disability or borderline intellect in females. Longitudinal observation in our patients further suggests that the clinical course may follow non-linear developmental trajectories with periods of arrest, regression, and partial recovery or improvement. However, all patients had intellectual disability to a different extent, which might reflect variation in severity or expression of the gene mutation within a shared clinical framework.

The key clinical contributions of our study could be:

- cerebral palsy–like motor phenotypes with fluctuating tone,
- severe neurodevelopmental impairment in the absence of characteristic neuroimaging findings,
- non-linear developmental trajectories not strictly associated with epilepsy,
- intrafamilial variability supporting variant interpretation.

In our cohort, pathogenic CASK variants were identified in 2% (2/105) of children undergoing targeted next-generation sequencing for suspected genetic neurodevelopmental disorders (CentoNeuro panel), a frequency comparable to previously reported rates of approximately 1–1.5% in selected cohorts with X-linked intellectual disability associated with microcephaly or congenital nystagmus, as well as in cohorts characterized by combined epilepsy and intellectual disability (6, 13). Bertoli-Avella et al. (14) reported an overall diagnostic yield of 32%–37% for their large-scale panel (CentoNeuro), with a 28%–31% yield for intellectual disability, validating the panel’s clinical utility over smaller tests.

In contrast, only three patients with CASK-related disorders were identified among approximately 6,179 patients evaluated in a pediatric neurology center over a 10-year period, supporting the notion that CASK-related disorders are substantially underrecognized in unselected clinical populations. The most specific neurological phenotype is MICPCH in females, and until the application of the CentoNeuro NGS panel in our study, predominantly these cases were suspected of possible CASK-related disorders. Therefore, other cases with neurodevelopmental delay or cerebral palsy–like phenotype without either microcephaly or cerebellar hypoplasia could be missed, and the real occurrence would be higher in pediatric neurology practice.

Molecular heterogeneity further contributes to diagnostic complexity. According to recent reviews, approximately 150 cases of CASK-related disorders have been reported to date, with nearly one quarter attributed to copy number variations (2, 3). This observation underscores the importance of diagnostic strategies incorporating next-generation sequencing with integrated CNV analysis, as demonstrated by studies reporting increased diagnostic yield in neurodevelopmental disorder cohorts (10, 11, 15).

The CASK c.1466G>A (p.Arg489Gln) variant was previously reported as a variant of uncertain significance among 100 consecutive patients with both epilepsy and intellectual disability diagnosed by exome sequencing (13). The reported case had limited phenotypic annotation, with severe intellectual disability, epilepsy with frequent seizures, and normal MRI, without follow-up or additional family data (13). According to recent ClinVar data, this variant is considered likely pathogenic, with few reports not described clinically in the literature (16. ClinVar; VCV000810578.68. Available at: https://www.ncbi.nlm.nih.gov/clinvar/variation/VCV000810578.68.) Our study provides the first detailed clinical and familial characterization of five cases with this variant, CASK c.1466G>A (p.Arg489Gln), supporting its pathogenicity and strengthening the existing classification as likely pathogenic.

Developmental delay (DD) and intellectual disability (ID) are the most constant features of CASK-related disorders, occurring with or without brain malformation and microcephaly, in both sexes. The severity of DD/ID is highly variable; in Giacomini’s group, it was moderate in 4/34, severe in 22/34, and profound in 8/34 cases (8). The phenotypic variability observed in our patients aligns with the current understanding of CASK protein function. CASK is a ubiquitously expressed multidomain scaffolding protein with particularly high expression in the brain, where it plays a critical role in synaptic organization, neuronal signaling, and regulation of genes involved in cortical development (5, 7). Disruption of distinct functional domains—through frameshift or nonsense variants leading to loss of function, or missense variants exerting hypomorphic or functional loss-of-function effects—provides a molecular basis for the wide clinical spectrum observed (5, 17).

Importantly, emerging experimental and clinical data support a neurodegenerative component in *CASK*-related disorders. *In vitro* studies have demonstrated that *CASK* mutations induce neurodegeneration and apoptosis of cerebellar granule cells, implicating disrupted CASK–Liprin-α interactions in neuronal loss (18). Additional evidence indicates that CASK plays a role in oxidative stress–induced microglial cell death via mitochondrial dysfunction and PARP-1 hyperactivation, suggesting mechanistic links between CASK dysfunction, neuroinflammation, and neurodegeneration (19). Furthermore, complete loss of CASK in males has been associated with progressive cerebellar degeneration, supporting the concept that *CASK*-related disorders may represent neurodegenerative conditions, particularly in hemizygous males (20). Consistent with these observations, all three patients in our cohort demonstrated developmental delay followed by periods of developmental arrest and/or regression, albeit with distinct age-dependent patterns. Similar phenomena have been rarely reported, including early childhood regression and late-onset motor regression during adolescence or adulthood (3, 4, 21).

Developmental regression has been described across a wide range of neurodevelopmental and epileptic conditions, including autism spectrum disorders and developmental epileptic encephalopathies; however, in our patients, developmental arrest or regression occurred before the onset of epilepsy in Patients 1 and 3 and in the absence of epilepsy in Patient 2. In Patient 1 and Patient 2, this coincided with the development of autistic features, whereas no clear association was observed in Patient 3. Based on these findings, it could be speculated that *CASK*-related disorders may be characterized by age-dependent vulnerability of neuronal networks, rather than representing static encephalopathies or epilepsy-driven regression alone. Further research with follow-up of more patients with *CASK*-related neurodevelopmental disorders, including longitudinal neuroimaging, biomarker evidence, or neuropathological data demonstrating progressive neuronal loss, is needed to support this hypothesis.

A clinically relevant and consistent feature across our cohort was the presence of abnormal muscle tone, supporting recent observations that tone dysregulation is a frequent but underrecognized component of *CASK*-related disorders (3). Hypotonia, hypertonia, or a combination of both (central hypotonia and hypertonia of the extremities) are suggested features of CASK-related disorders, as described by Moog and Kutsche (2). Muscle tone abnormalities were also described in most patients (predominantly hypotonia, but also limb hypertonia) (22). In Giacomini’s series, the main neurological findings included generalized hypotonia in 67.6% (23/34) of cases, distal limb hypertonia either isolated in 47% (16/34) or associated with dystonia in 20.6% (7/34) of patients, and ataxia in 55.9% (19/34). Two of our patients exhibited axial hypotonia combined with either limb spasticity or variable muscle tone and intermittent athetoid movements, resulting in mixed cerebral palsy–like phenotypes, while the third patient showed predominantly axial hypotonia with a clumsy gait resembling an ataxic cerebral palsy phenotype. The fluctuating and progressive nature of these motor abnormalities distinguishes *CASK*-related disorders from static cerebral palsy and supports their classification as cerebral palsy mimics, warranting genetic evaluation when atypical features are present. We have not encountered in the literature a *CASK*-related disorder explicitly described as a cerebral palsy imitator, but our cases, together with many of those described in the literature, support the statement that *CASK*-related disorders could present as a cerebral palsy mimic.

Recent large-scale reviews, including Martin et al. (3), have primarily compared patients with MICPCH and those with microcephaly without pontocerebellar hypoplasia, noting that only a small number of reported individuals lacked both microcephaly and structural brain abnormalities. In contrast, Patient 3 in our series exhibited neither microcephaly nor pontocerebellar malformations, yet presented with a severe neurodevelopmental phenotype, including profound intellectual disability, severe motor impairment, drug-resistant epilepsy, and autistic features. Another reported case with the same *CASK* variant had a similar clinical picture and normal MRI (13). This observation supports the currently described broad spectrum and indicates that severe functional impairment may occur in the absence of microcephaly or major structural brain anomalies, limiting the prognostic value of neuroimaging findings alone.

Autism spectrum–related features represented another important and clinically relevant aspect of CASK-related disorders in our cohort. Consistent with recent systematic assessments demonstrating elevated autism-related traits in individuals with CASK variants (3), all three patients exhibited autistic manifestations, ranging from isolated motor stereotypies in Patient 2 to severe social-communication impairment meeting criteria for autism spectrum disorder in the other two patients. Notably, autistic features were often more pronounced following periods of developmental regression and, in two cases, co-occurred with epilepsy, suggesting shared underlying synaptic and network-level dysfunction. These findings reinforce the concept of CASK as a synaptopathy, with psychiatric manifestations representing a core component of the disease spectrum rather than secondary comorbidities (5).

Epilepsy was present in two of the three patients and was characterized by onset after early childhood and pharmacoresistance, consistent with previous reports describing refractory epilepsy in CASK-related disorders, particularly in males (3, 5, 8). Epileptic spasms occurred in 1 of our 3 patients, similar to the 32.3% reported by Giacomini et al. (8). These authors suggest serial and systematic monitoring because of possible worsening of epilepsy over time. They also concluded that epilepsy is not associated with more severe developmental disability (there was no difference between non-epileptic patients and those with generalized seizures or spasms), in contrast to Martin et al. (3), who reported that epilepsy is significantly associated with intellectual disability severity after controlling for age within the BINGO CASK-related group. Our data are in accordance with Martin’s conclusion: two of our three patients had epilepsy and both had profound intellectual disability at the current age, in contrast to moderate intellectual disability in the patient without epilepsy, although she is only 4 years of age. Notably, developmental arrest or regression in our cohort was not linked to epilepsy onset (the latter started years after the arrest or regression, and there was no epilepsy in Patient 2 with speech regression and only partial recovery), further supporting the role of broader neurobiological mechanisms beyond seizure activity alone.

Ophthalmological abnormalities were variable in our cohort. Nystagmus was observed in only one patient, whereas strabismus and partial optic atrophy represented more consistent ocular findings. Our results are similar to those reported by Giacomini et al. (8) (strabismus in 38.2% [13/34], nystagmus in 32.4% [11/34], and optic atrophy in 29.4% [10/34] of cases) and by Martin et al. (3) (optic atrophy 28.6%, strabismus 30.4%, and nystagmus 25%). Although ocular manifestations have been reported as diagnostic clues in CASK-related disorders (2, 6), they were not a dominant feature in our patients or in other reported cases.

Hearing impairment could not be systematically evaluated, as auditory evoked potentials were performed in only one patient; therefore, no definitive conclusions regarding auditory involvement can be drawn. However, according to the literature, sensorineural hearing loss is present in approximately one quarter to one third of patients with CASK mutations (1, 3).

Data from the BINGO CASK project revealed a consistent prevalence of tone abnormalities (as in our series), epilepsy, and sensorineural hearing loss, but a lower prevalence of severe/profound intellectual disability, optic atrophy, and nystagmus (3).

A consistent and notable finding across all patients in our cohort was postnatal growth retardation, regardless of sex, genotype, or neuroimaging phenotype. Although growth impairment has been sporadically reported in CASK-related disorders, it has not been systematically emphasized as a core clinical feature (1, 3, 4). Experimental data demonstrate that CASK regulates postnatal brain growth and mitochondrial oxidative metabolism, while its widespread expression in multiple organs may further contribute to impaired somatic growth (4, 5, 23). Our findings suggest that unexplained postnatal growth failure, particularly when accompanied by neurodevelopmental delay and a cerebral palsy–like neurological phenotype or ophthalmological abnormalities, should raise clinical suspicion for CASK-related disorders.

Although dysmorphic features such as retrognathia, fleshy uplifted earlobes, high nasal bridge, upslanting palpebral fissures, long convex fingernails, and overriding second toes have been described in CASK-related disorders (6, 22), these features are highly variable and not specific for this genetic condition, as in our series. Therefore, they should be regarded as non-specific diagnostic clues rather than defining features.

Finally, the marked intrafamilial phenotypic variability observed in the multigenerational family, including among males carrying the same CASK variant (a continuum from severe phenotype with intellectual disability, severe epilepsy, and no MRI anomaly to mild intellectual disability and clumsy gait without epilepsy until 37 years of age), underscores the limited predictive value of genotype alone. In males, three phenotypic groups have been previously described: (i) MICPCH with severe epileptic encephalopathy caused by hemizygous loss-of-function mutations, (ii) MICPCH associated with inactivating alterations in the mosaic state or a partly penetrant mutation, and (iii) syndromic/nonsyndromic mild to severe intellectual disability with or without nystagmus caused by CASK missense and splice mutations that leave the CASK protein intact but likely alter its function or reduce the amount of normal protein (22). According to this phenotypic categorization, the males in Patient 3’s family should be considered as having syndromic/nonsyndromic mild to severe intellectual disability with or without nystagmus. We suggest that another phenotype category may be considered: mild to severe intellectual disability with a cerebral palsy–like phenotype. Moog et al. (22) recommend CASK testing in male patients with the combination of developmental delay/intellectual disability or epileptic encephalopathy, postnatal microcephaly (< −3 SD), and pontocerebellar hypoplasia, and also consider sequencing of exons 22–27 in patients with intellectual disability and nystagmus. In contrast, based on our data, we suggest that CASK-related disorders should also be suspected in males with intellectual disability and a cerebral palsy–like phenotype, with or without epilepsy.

Missense CASK mutations are usually hypomorphic, leading to reduced protein function. There are no definitive data in ClinVar confirming that the missense mutation in Patient 3 is a hypomorphic variant; however, based on the clinical presentation of all affected family members and considering the genotype–phenotype correlations described by Moog et al. (22), this variant, CASK c.1466G>A (p.Arg489Gln), could be considered hypomorphic. Notably, female carriers of this missense variant in the family of Patient 3 exhibited only mild to borderline intellectual disability without additional neurological manifestations, similar to other reported females with missense variants. Such subtle phenotypes may be easily overlooked in the absence of genetic testing, emphasizing that CASK-related disorders should be considered in females with unexplained mild intellectual disability or borderline intellect, particularly in families with X-linked patterns of neurodevelopmental disorders. These observations suggest the influence of additional genetic, epigenetic, metabolic, or environmental modifiers and highlight the need for further studies to elucidate the molecular mechanisms underlying clinical heterogeneity in CASK-related disorders.

### Limitations

This study has several limitations. First, the small number of index patients (n = 3) limits the generalizability of the findings. Second, the retrospective design and the use of selected subgroups (e.g., patients undergoing targeted genetic testing) introduce potential selection bias. In particular, the increased availability of next-generation sequencing and CNV analysis in the later years of the study period (2023–2024) may have influenced case detection.

Third, although additional affected relatives were identified and partially clinically evaluated, detailed and standardized clinical data were not available for all family members, and neuroimaging data were lacking. This limited the extent of systematic intrafamilial phenotypic characterization.

Finally, the absence of longitudinal neuroimaging or biomarker data limits interpretation of developmental regression and precludes definitive conclusions regarding potential neurodegenerative mechanisms.

Despite these limitations, the inclusion of a multigenerational family provides additional insight into intrafamilial variability in CASK-related disorders.

## Conclusion

This study expands the clinical spectrum of *CASK*-related neurodevelopmental disorders by presenting the first reported cases from Bulgaria and providing evidence of intra- and interfamilial phenotypic variability. Our findings suggest that *CASK*-related disorders may present with cerebral palsy–like motor phenotypes, non-linear developmental trajectories, and severe neurodevelopmental impairment even in the absence of characteristic neuroimaging abnormalities. Consistent postnatal growth retardation observed across our cohort may represent an underrecognized clinical feature of these disorders.

Our findings underscore the importance of comprehensive genetic testing, including next-generation sequencing in patients with unexplained neurodevelopmental disorders, syndromic and non-syndromic intellectual disability, postnatal growth retardation, atypical cerebral palsy phenotypes, pharmacoresistant epilepsy, and additional autistic or ocular features. Individuals—particularly females with mild intellectual disability—may be easily overlooked without comprehensive gene panel testing that includes *CASK*, suggesting that *CASK*-related disorders remain substantially underdiagnosed.

Increased clinical awareness and the use of comprehensive genetic testing are essential for accurate diagnosis, appropriate genetic counseling, and improved clinical management of affected individuals.

Further studies are needed to clarify the molecular mechanisms underlying *CASK* dysfunction and the factors contributing to marked clinical heterogeneity, which may ultimately inform future targeted therapeutic approaches.

## Data Availability

The original contributions presented in the study are included in the article/**Supplementary Material**. Further inquiries can be directed to the corresponding author.

## Supplementary material

Supplementary Table 1 List of genes included in the CentoNeuro panel.
