# Supplementary material for: Clinical heterogeneity and diagnostic challenges in CASK-related neurodevelopmental disorders: a longitudinal observational study
Source: Front Psychiatry. 2026 Jul 13;17:1804756. doi: 10.3389/fpsyt.2026.1804756 (PMC13403493; doi:10.3389/fpsyt.2026.1804756)
Supplement: Supplementary Table 1 — List of genes included in the CentoNeuro panel. [file Table1.docx]

Supplementary Table 1. List of genes included in the CentoNeuro panel **(sequencing and NGS-based CNV analyses), CENTOGENE GmbH •**

*AAAS, AARS1, AARS2, AASS, ABAT, ABCA1, ABCA7, ABCB6, ABCB7, ABCC6, ABCC8, ABCD1, ABCD3, ABCD4, ABHD12, ABHD5, ACACA, ACAD8, ACAD9, ACADM, ACADS, ACADSB, ACADVL, ACAT1, ACE, ACHE, ACO2, ACOX1, ACSF3, ACSL4, ACTA1, ACTA2, ACTB, ACTG1, ACTG2, ACTL6B, ACTN4, ACVRL1, ACY1, ADA, ADAM10, ADAM22, ADAMTS10, ADAMTSL2, ADAR, ADAT3, ADCY5, ADGRG1, ADGRG6, ADGRV1, ADK, ADNP, ADPRS, ADSL, AFF2, AFF3, AFG3L2, AGA, AGK, AGL, AGPS, AGRN, AGTPBP1, AGXT, AHCY, AHDC1, AHI1, AIFM1, AIMP1, AIMP2, AK2, AKT3, ALAD, ALAS2, ALDH18A1, ALDH2, ALDH3A2, ALDH4A1, ALDH5A1, ALDH6A1, ALDH7A1, ALDOA, ALDOB, ALG1, ALG11, ALG12, ALG13, ALG14, ALG2, ALG3, ALG6, ALG8, ALG9, ALPL, ALS2, ALX1, ALX3, ALX4, AMACR, AMMECR1, AMPD1, AMPD2, AMT, ANG, ANK2, ANK3, ANKLE2, ANKRD11, ANO10, ANO3, ANO5, ANTXR2, ANXA11, AP1S1, AP1S2, AP2M1, AP3B1, AP3B2, AP4B1, AP4E1, AP4M1, AP4S1, AP5Z1, APOE, APP, APTX, ARFGEF2, ARG1, ARHGAP31, ARHGEF10, ARHGEF6, ARHGEF9, ARID1A, ARID1B, ARID2, ARL13B, ARL6, ARL6IP1, ARSA, ARSB, ARSL, ARV1, ARX, ASAH1, ASCC1, ASCL1, ASH1L, ASL, ASNS, ASPA, ASPM, ASS1, ASTN2, ASXL1, ASXL3, ATAD1, ATCAY, ATIC, ATL1, ATM, ATN1, ATP13A2, ATP1A1, ATP1A2, ATP1A3, ATP2A1, ATP2A2, ATP2B3, ATP2B4, ATP5F1A, ATP5F1E, ATP6AP1, ATP6AP2, ATP6V0A2, ATP6V1A, ATP7A, ATP7B, ATP8A2, ATPAF2, ATR, ATRX, AUH, AUTS2, B3GALNT2, B3GLCT, B4GALNT1, B4GALT1, B4GAT1, B9D1, B9D2, BAG3, BBS1, BBS10, BBS12, BBS2, BBS4, BBS5, BBS7, BBS9, BCAP31, BCKDHA, BCKDHB,BCKDK, BCL11A, BCOR, BCS1L, BDNF, BEST1, BICD2, BIN1, BLOC1S1, BLOC1S3, BLOC1S6, BOLA3, BRAF, BRAT1, BRWD3, BSCL2, BSND, BTD, BVES, C12ORF4, C12orf57, C19orf12,C1QBP, CA2, CA5A, CA8, CACNA1A, CACNA1B, CACNA1C, CACNA1D, CACNA1E, CACNA1F, CACNA1G, CACNA1H, CACNA1S, CACNA2D2, CACNB2, CACNB4, CAD, CAMK2A, CAMK2B,CAMK2G, CAMTA1, CAPN1, CAPN3, CARD11, CARS2, CASK, CASQ1, CASR, CAT, CAV1, CAV3, CAVIN1, CBL, CBS, CC2D1A, CC2D2A, CCDC115, CCDC22, CCDC40, CCDC78, CCDC88A,CCDC88C, CCM2, CCNF, CCT5, CD320, CD59, CD96, CDH11, CDH15, CDK5RAP2, CDKL5, CDON, CEL, CENPF, CENPJ, CEP135, CEP152, CEP164, CEP290, CEP41, CEP63, CERS1,CERT1, CFAP418, CFL2, CHAMP1, CHAT, CHCHD10, CHCHD2, CHD1, CHD2, CHD3, CHD7, CHD8, CHKB, CHL1, CHMP1A, CHMP2B, CHRNA1, CHRNA2, CHRNA4, CHRNA7, CHRNB1, CHRNB2, CHRND, CHRNE, CHRNG, CHST14, CHSY1, CIB2, CIC, CILK1, CISD2, CIT, CLCN1, CLCN2, CLCN4, CLCNKA, CLCNKB, CLDN16, CLDN19, CLN3, CLN5, CLN6, CLN8, CLP1,CLPB, CLPP, CLTC, CNBP, CNGB3, CNKSR2, CNNM2, CNPY3, CNTN4, CNTNAP1, CNTNAP2, COA5, COA6, COA7, COA8, COASY, COG1, COG4, COG5, COG6, COG7, COG8, COL11A2,COL12A1, COL13A1, COL2A1, COL4A1, COL4A2, COL6A1, COL6A2, COL6A3, COLGALT1, COLQ, COMT, COQ2, COQ4, COQ6, COQ7, COQ8A, COQ8B, COQ9, COX10, COX14, COX15,COX20, COX4I2, COX6A1, COX6B1, COX7B, CP, CPA6, CPLANE1, CPLX1, CPOX, CPS1, CPT1A, CPT1C, CPT2, CRADD, CRBN, CREBBP, CRIPT, CRLF1, CRPPA, CRYAB, CSF1R, CSMD1,CSNK2B, CSPP1, CSRP3, CST3, CSTB, CTC1, CTCF, CTDP1, CTNNA2, CTNNA3, CTNNB1, CTNS, CTSA, CTSC, CTSD, CTSF, CTSK, CUL3, CUL4B, CUL7, CUX1, CUX2, CWF19L1, CX3CR1,CYB5A, CYB5R3, CYC1, CYCS, CYFIP2, CYLD, CYP11A1, CYP11B1, CYP11B2, CYP24A1, CYP27A1, CYP27B1, CYP2U1, CYP7B1, D2HGDH, DAB1, DAG1, DARS1, DARS2, DBT, DCAF17, DCTN1, DCX, DDC, DDHD1, DDHD2, DDOST, DDX3X, DEAF1, DEGS1, DENND5A, DEPDC5, DES, DGUOK, DHCR24, DHCR7, DHDDS, DHFR, DHH, DHODH, DHPS, DHTKD1, DHX30,DIABLO, DIAPH1, DIAPH3, DIP2B, DKC1, DLAT, DLD, DLG3, DLG4, DLGAP2, DLL3, DLX3, DMD, DMGDH, DMPK, DMXL2, DNA2, DNAJB2, DNAJB6, DNAJC12, DNAJC13, DNAJC19,DNAJC5, DNAJC6, DNM1, DNM1L, DNM2, DNMT1, DNMT3A, DOCK3, DOCK6, DOCK7, DOCK8, DOK7, DOLK, DPAGT1, DPF2, DPM1, DPM2, DPM3, DPP6, DPYD, DPYS, DRD3, DST,DSTYK, DTNBP1, DVL3, DYM, DYNC1H1, DYNC2H1, DYRK1A, DYSF, EARS2, EBF3, EBP, ECEL1, ECHS1, EDC3, EDN3, EDNRB, EEF1A2, EFHC1, EFTUD2, EGF, EGR2, EHMT1, EIF2B1,EIF2B2, EIF2B3, EIF2B4, EIF2B5, EIF2S3, EIF3F, EIF4G1, ELAC2, ELOVL4, ELOVL5, ELP1, ELP2, EMC10, EMD, EML1, EMX2, ENO3, ENTPD1, EP300, EPB41L1, EPG5, EPHX2, EPM2A,EPRS1, ERBB4, ERCC1, ERCC2, ERCC5, ERCC6, ERCC8, ERLIN1, ERLIN2, ESCO2, ETFA, ETFB, ETFDH, ETHE1, EWSR1, EXOC6B, EXOSC3, EXOSC8, EXOSC9, EXT1, EZH2, F2, F5, FA2H,FADD, FAH, FAM126A, FAN1, FANCB, FARS2, FARSB, FASTKD2, FAT2, FBLN5, FBN1, FBN2, FBXL4, FBXO11, FBXO38, FBXO7, FDX2, FDXR, FECH, FEZF1, FGA, FGD1, FGD4, FGF10, FGF12, FGF14, FGFR2, FGFR3, FH, FHL1, FIG4, FKBP10, FKBP14, FKRP, FKTN, FLAD1, FLNA, FLNC, FLVCR1, FLVCR2, FMN2, FOLR1, FOXC1, FOXG1, FOXL2, FOXP1, FOXP2, FOXRED1,FRMD7, FRMPD4, FRRS1L, FTL, FTO, FTSJ1, FUCA1, FUS, FUT8, FXN, FXR1, FXYD2, G6PD, GAA, GABBR2, GABRA1, GABRA2, GABRA5, GABRB1, GABRB2, GABRB3, GABRD, GABRE,GABRG2, GAD1, GALC, GALNS, GALT, GAMT, GAN, GARS1, GATAD2B, GATM, GBA, GBA2, GBE1, GCDH, GCH1, GCK, GCSH, GDAP1, GDI1, GDNF, GFAP, GFER, GFM1, GFM2, GFPT1,GIGYF2, GJA1, GJB1, GJB3, GJC2, GK, GLA, GLB1, GLDC, GLDN, GLE1, GLI2, GLI3, GLO1, GLRA1, GLRB, GLRX5, GLUD1, GLUL, GLYCTK, GM2A, GMPPA, GMPPB, GNAL, GNAO1, GNAQ,GNAS, GNB1, GNB4, GNB5, GNE, GNPAT, GNPTAB, GNPTG, GNS, GOSR2, GOT2, GPAA1, GPC3, GPC4, GPC6, GPHN, GPI, GPT2, GPX1, GRHPR, GRIA1, GRIA2, GRIA3, GRIA4, GRID2,GRIK2, GRIN1, GRIN2A, GRIN2B, GRIN2D, GRIP1, GRM1, GRN, GSN, GSR, GSS, GTPBP2, GTPBP3, GUF1, GUSB, GYG1, GYS1, HACE1, HADH, HADHA, HADHB, HAMP, HARS2, HAX1,HBB, HCCS, HCFC1, HCN1, HDAC4, HDAC8, HECW2, HEPACAM, HERC2, HESX1, HEXA, HEXB, HGSNAT, HIBCH, HIKESHI, HINT1, HIVEP2, HK1, HLCS, HMBS, HMGCL, HMGCS2, HNMT, HNRNPA1, HNRNPA2B1, HNRNPDL, HNRNPH2, HNRNPR, HNRNPU, HOGA1, HOXA1, HOXD10, HPCA, HPD, HPRT1, HPS1, HPS4, HPS5, HPS6, HRAS, HSD11B1, HSD17B10,HSD17B4, HSD3B2, HSPA9, HSPB1, HSPB3, HSPB8, HSPD1, HSPG2, HTRA1, HTRA2, HUWE1, HYAL1, HYDIN, IARS2, IBA57, IDH2, IDH3B, IDS, IDUA, IER3IP1, IFIH1, IFT140, IFT172,IFT27, IGBP1, IGF1, IGF1R, IGHMBP2, IL1RAPL1, IMPA1, INF2, INPP5E, INVS, IQSEC2, IRF2BPL, IRX5, ISCA1, ISCA2, ISCU, ITGA7, ITGB3, ITM2B, ITPA, ITPR1, IVD, JAG1, JAM2, JAM3,KANK1, KANSL1, KARS1, KAT6A, KAT6B, KAT8, KATNB1, KBTBD13, KCNA1, KCNA2, KCNB1, KCNC1, KCNC3, KCND3, KCNE3, KCNH1, KCNJ1, KCNJ10, KCNJ2, KCNK18, KCNK4,KCNK9, KCNMA1, KCNQ2, KCNQ3, KCNQ5, KCNT1, KCNT2, KCTD17, KCTD3, KCTD7, KDM4B, KDM5B, KDM5C, KDM6A, KIAA1109, KIDINS220, KIF11, KIF14, KIF1A, KIF1B, KIF1C,KIF21A, KIF2A, KIF5A, KIF5C, KIF7, KIFBP, KIRREL3, KLHL40, KLHL41, KLHL7, KMT2A, KMT2B, KMT2C, KMT2D, KMT2E, KMT5B, KNL1, KPTN, KRAS, KRIT1, KRT5, KRT8, KY, L1CAM,L2HGDH, LAMA1, LAMA2, LAMB1, LAMB2, LAMC3, LAMP2, LARGE1, LARS2, LAT, LBR, LDB3, LDHA, LEP, LGI1, LGI4, LHX3, LHX4, LIAS, LIMS2, LINS1, LIPA, LIPT1, LIPT2, LITAF, LMAN2L, LMBRD1, LMNA, LMNB1, LMOD3, LMX1B, LONP1, LPIN1, LRBA, LRP1, LRP10, LRP2, LRP4, LRPPRC, LRRK2, LRSAM1, LYRM7, LYST, LZTFL1, LZTR1, MACF1, MAF, MAG,MAGEL2, MAGI2, MAGT1, MAMLD1, MAN1B1, MAN2B1, MANBA, MAOA, MAP2K1, MAP2K2, MAPT, MARS1, MARS2, MASP1, MATR3, MBD5, MBOAT7, MBTPS2, MCCC1, MCCC2,MCEE, MCM4, MCM6, MCOLN1, MCPH1, MDH2, MECP2, MECR, MED12, MED13, MED13L, MED17, MED23, MED25, MEF2C, MEGF10, MEIS2, METTL23, MFF, MFN2, MFRP, MFSD2A,MFSD8, MGAT2, MGME1, MIB1, MICU1, MID1, MIPEP, MITF, MKKS, MKS1, MLC1, MLPH, MLYCD, MMAA, MMAB, MMACHC, MMADHC, MMUT, MOCS1, MOCS2, MOGS, MPC1,MPDU1, MPDZ, MPI, MPV17, MPZ, MRE11, MRPL3, MRPL44, MRPS16, MRPS2, MRPS22, MRPS34, MSMO1, MSR1, MSRB3, MSTO1, MSX1, MSX2, MTFMT, MTHFD1, MTHFR, MTHFS,MTM1, MTMR14, MTMR2, MTO1, MTOR, MTPAP, MTR, MTRFR, MTRR, MTTP, MUSK, MYBPC1, MYBPC3, MYCN, MYH2, MYH3, MYH7, MYH8, MYL1, MYL2, MYMK, MYO18B, MYO1E,MYO5A, MYO9A, MYO9B, MYORG, MYOT, MYPN, MYT1L, NAA10, NAA15, NACC1, NADK2, NAGA, NAGLU, NAGS, NALCN, NARS2, NAXD, NAXE, NBAS, NBEA, NCAPD3, NDE1, NDP, NDRG1, NDST1, NDUFA1, NDUFA10, NDUFA11, NDUFA12, NDUFA2, NDUFA6, NDUFA9, NDUFAF1, NDUFAF2, NDUFAF3, NDUFAF4, NDUFAF5, NDUFAF6, NDUFB11, NDUFB3,NDUFB8, NDUFB9, NDUFS1, NDUFS2, NDUFS3, NDUFS4, NDUFS6, NDUFS7, NDUFS8, NDUFV1, NDUFV2, NEB, NECAP1, NECTIN1, NEDD4L, NEFH, NEK1, NEK10, NEU1, NEUROD2, NEXMIF, NF1, NFE2L2, NFIA, NFIB, NFIX, NFU1, NGF, NGLY1, NHEJ1, NHLRC1, NHS, NIPA1, NIPBL, NKX6-2, NLGN3, NLGN4X, NLRP12, NLRP3, NNT, NOTCH1, NOTCH2, NOTCH3, NPC1, NPC2, NPHP1, NPHP3, NPR2, NPRL2, NPRL3, NR2F1, NR3C2, NRXN1, NSD1, NSD2, NSDHL, NSMCE3, NSUN2, NT5C2, NTHL1, NTRK1, NTRK2, NUBPL, NUP133, NUP62, NUS1, OAT, OCLN, OCRL, ODAD4, OFD1, OGDH, OPA1, OPA3, OPHN1, OPTN, ORC1, OSGEP, OTC, OTUD6B, OXCT1, P4HA2, P4HB, P4HTM, PACS1, PACS2, PAFAH1B1, PAH, PAK1, PAK3, PAM16, PANK2, PARK7, PARS2, PAX3, PAX6, PAX7, PBX1, PC, PCBD1, PCCA, PCCB, PCDH12, PCDH15, PCDH19, PCK2, PCNT, PCYT2, PDCD1, PDCD10, PDE10A, PDE6D, PDE8B, PDGFB, PDGFRB, PDHA1, PDHB, PDHX, PDK3, PDP1, PDSS1, PDSS2, PDX1, PDYN, PER2, PET100, PEX1, PEX10, PEX11B, PEX12, PEX13, PEX14, PEX16, PEX19, PEX2, PEX26, PEX3, PEX5, PEX6, PEX7, PFKM, PFN1, PGAM2, PGAP1, PGAP2, PGK1, PGM1, PHACTR1, PHF6, PHF8, PHGDH, PHIP, PHKA1, PHOX2B, PHYH, PIEZO2, PIGA, PIGB, PIGC, PIGG, PIGH, PIGL, PIGN, PIGO, PIGP, PIGQ, PIGS, PIGT, PIGU, PIGV, PIGW, PIK3CA, PIK3R2, PIK3R5, PINK1, PIP5K1C, PITX1, PITX2, PKLR, PLA2G6, PLAA, PLCB1, PLCG2, PLEC, PLEKHG2, PLEKHG5, PLK1, PLK4, PLN, PLOD2, PLP1, PLPBP, PLXNB3, PMM2, PMP22, PMPCA, PMPCB, PNKD, PNKP, PNPLA2, PNPLA6, PNPLA8, PNPO, PNPT1, POGLUT1, POGZ, POLA1, POLG, POLG2, POLR1C, POLR1D, POLR3A, POLR3B, POMGNT1, POMGNT2, POMK, POMT1, POMT2, PON1, POP1, PORCN, POT1, POU1F1, PPM1D, PPOX, PPP2CA, PPP2R1A, PPP2R5D, PPP3CA, PPT1, PQBP1, PREPL, PRF1, PRICKLE1, PRICKLE2, PRKAG2, PRKCA, PRKCG, PRKN, PRKRA, PRMT7, PRNP, PRODH, PROP1, PRPH, PRPS1, PRRT2, PRRX1, PRSS12, PRX, PSAP, PSAT1, PSEN1, PSEN2, PSMD12, PSPH, PTCH1, PTCHD1, PTEN, PTF1A, PTPN11, PTPN23, PTPRC, PTRH2, PTS, PUM1, PURA, PUS1, PUS3, PXDN, PYCR1, PYCR2, PYGM, PYROXD1, QARS1, QDPR, QRICH1, RAB11A, RAB11B, RAB18, RAB27A, RAB39B, RAB3GAP1, RAB3GAP2, RAB7A, RAC1, RAD21, RAD50, RAF1, RAI1, RALA, RALGAPA1, RAP1GDS1, RAPSN, RARS1, RARS2, RBBP8, RBCK1, RBFOX1, RBM10, RBM8A, RDH11, REEP1, REEP2, RELN, RERE, REST, RET, RETREG1, RFT1, RHOBTB2, RIMS1, RIN2, RMND1, RNASEH1, RNASEH2A, RNASEH2B, RNASEH2C, RNASET2, RNF113A, RNF13, RNF135, RNF168, RNF170, RNF216, ROBO2, ROGDI, ROR2, RORA, RORB, RPGRIP1L, RPIA, RPL10, RPL35A, RPS14, RPS6KA3, RRM2B, RTN2, RTN4IP1, RTTN, RUBCN, RUSC2, RXYLT1, RYR1, SACS, SALL1, SAMD9L, SAMHD1, SARS2, SASH1, SASS6, SATB2, SBDS, SBF1, SBF2, SC5D, SCARB2, SCN10A, SCN1A, SCN1B, SCN2A, SCN3A, SCN4A, SCN8A, SCN9A, SCO1, SCO2, SCYL1, SDCCAG8, SDHA, SDHAF1, SDHAF2, SDHB, SDHD, SEC23B, SECISBP2, SELENOI, SELENON, SEMA5A, SEMA6B, SEPSECS, SERAC1, SERPINI1, SET, SETBP1, SETD1A, SETD2, SETD5, SETX, SF3B1, SFXN4, SGCA, SGCB, SGCD, SGCE, SGCG, SGSH, SH3TC2, SHANK2, SHH, SHOC2, SHROOM4, SIGMAR1, SIK1, SIL1, SIN3A, SIX3, SKI, SLC12A3, SLC12A5, SLC12A6, SLC13A3, SLC13A5, SLC16A1, SLC16A2, SLC17A5, SLC18A3, SLC19A2, SLC19A3, SLC1A1, SLC1A2, SLC1A3, SLC1A4, SLC20A2, SLC22A5, SLC25A1, SLC25A12, SLC25A13, SLC25A15, SLC25A19, SLC25A20, SLC25A22, SLC25A26, SLC25A3, SLC25A38, SLC25A4, SLC25A42, SLC25A46, SLC27A4, SLC2A1, SLC2A10, SLC30A10, SLC33A1, SLC35A1, SLC35A2, SLC35A3, SLC35C1, SLC39A14, SLC39A8, SLC3A1, SLC4A10, SLC4A4, SLC52A2, SLC52A3, SLC5A7, SLC6A1, SLC6A17, SLC6A19, SLC6A3, SLC6A4, SLC6A5, SLC6A8, SLC6A9, SLC7A7, SLC9A6, SLC9A9, SLCO1B3, SMAD4, SMARCA2, SMARCA4, SMARCB1, SMARCC2, SMARCE1, SMC1A, SMC3, SMCHD1, SMPD1, SMPD4, SMS, SNAI2, SNAP25, SNAP29, SNCA, SNCB, SNIP1, SNTA1, SNX14, SNX27, SOBP, SOD1, SOD2, SON, SORL1, SOS1, SOX10, SOX11, SOX2, SOX3, SOX5, SPART, SPAST, SPATA5, SPEG, SPG11, SPG21, SPG7, SPR, SPTAN1, SPTBN2, SPTBN4, SPTLC1, SPTLC2, SQSTM1, SRCAP, SRD5A3, SSR4, ST3GAL3, ST3GAL5, STAC3, STAG1, STAMBP, STAR, STAT1, STAT2, STIL, STIM1, STRA6, STRADA, STT3A, STUB1, STX1B, STXBP1, SUCLA2, SUCLG1, SUGCT, SUMF1, SUN2, SUOX, SURF1, SYN1, SYN2, SYNE1, SYNE2, SYNGAP1,*
